# TNFα Altered Inflammatory Responses, Impaired Health and Productivity, but Did Not Affect Glucose or Lipid Metabolism in Early-Lactation Dairy Cows

**DOI:** 10.1371/journal.pone.0080316

**Published:** 2013-11-19

**Authors:** Kai Yuan, Jaymelynn K. Farney, Laman K. Mamedova, Lorraine M. Sordillo, Barry J. Bradford

**Affiliations:** 1 Department of Animal Sciences and Industry, Kansas State University, Manhattan, Kansas, United States of America; 2 Department of Large Animal Clinical Sciences, Michigan State University, East Lansing, Michigan, United States of America; Van Andel Institute, United States of America

## Abstract

Inflammation may be a major contributing factor to peripartum metabolic disorders in dairy cattle. We tested whether administering an inflammatory cytokine, recombinant bovine tumor necrosis factor-α (rbTNFα), affects milk production, metabolism, and health during this period. Thirty-three Holstein cows (9 primiparous and 24 multiparous) were randomly assigned to 1 of 3 treatments at parturition. Treatments were 0 (Control), 1.5, or 3.0 µg/kg body weight rbTNFα, which were administered once daily by subcutaneous injection for the first 7 days of lactation. Statistical contrasts were used to evaluate the treatment and dose effects of rbTNFα administration. Plasma TNFα concentrations at 16 h post-administration tended to be increased (*P*<0.10) by rbTNFα administration, but no dose effect (*P*>0.10) was detected; rbTNFα treatments increased (*P*<0.01) concentrations of plasma haptoglobin. Most plasma eicosanoids were not affected (*P*>0.10) by rbTNFα administration, but 6 out of 16 measured eicosanoids changed (*P*<0.05) over the first week of lactation, reflecting elevated inflammatory mediators in the days immediately following parturition. Dry matter and water intake, milk yield, and milk fat and protein yields were all decreased (*P*<0.05) by rbTNFα treatments by 15 to 18%. Concentrations of plasma glucose, insulin, β-hydroxybutyrate, non-esterified fatty acids, triglyceride, 3-methylhistidine, and liver triglyceride were unaffected (*P*>0.10) by rbTNFα treatment. Glucose turnover rate was unaffected (*P* = 0.18) by rbTNFα administration. The higher dose of rbTNFα tended to increase the risk of cows developing one or more health disorders (*P* = 0.08). Taken together, these results indicate that administration of rbTNFα daily for the first 7 days of lactation altered inflammatory responses, impaired milk production and health, but did not significantly affect liver triglyceride accumulation or nutrient metabolism in dairy cows.

## Introduction

The periparturient period in dairy cows is characterized by substantial metabolic stress, endocrine changes, depressed feed intake, and negative energy balance [1]. In response to these changes, dairy cows mobilize adipose tissue triglyceride (TG), leading to elevated non-esterified fatty acid (NEFA) concentrations in the blood. The high flux of blood NEFA to the liver often exceeds the capacity of the liver to completely oxidize NEFA to CO_2_, resulting in partial oxidation to form ketones or esterification to form TG within hepatocytes [2]. Excessive production of ketones often leads to ketosis, which is characterized by elevated blood concentrations of β-hydroxybutyrate (BHBA), depressed appetite, and decreased milk production [3]. Furthermore, because ruminants are inefficient at exporting TG from the liver, excessive TG accumulation or fatty liver occurs. Ketosis and fatty liver affect up to 50% of dairy cows, compromising production, health, and reproduction [3], [4]. Gluconeogenesis is of great importance at all times in ruminants, providing up to 90% of the necessary glucose [5]. Meeting glucose needs can be a tremendous metabolic challenge for early-lactation dairy cows, which often experience inadequate feed intake. Although lipid and glucose metabolism in periparturient cows has been extensively studied, the mechanisms underlying the development of these metabolic disorders are not fully understood.

Inflammatory mediators play critical roles in immunity and metabolism, and recent research has suggested that inflammation is involved in metabolic disorders as well. For example, obesity is associated with a chronic low-grade inflammatory state in multiple metabolic tissues including adipose, liver, muscle, pancreas, and brain [6]. Compared with lean controls, obese mice had increased levels of adipose tissue tumor necrosis factor-α (TNFα), a potent cytokine capable of triggering inflammatory responses [7]. In dairy cows, fatty liver was associated with increased plasma inflammation markers, including haptoglobin and serum amyloid A [8]. Although a recent study reported that plasma concentrations of TNFα in periparturient cows were decreased postpartum compared with prepartum [9], elevated activity of serum TNFα was observed in cows with moderate to severe fatty liver [10]. In light of these findings, we hypothesized that chronic low-grade inflammation may be a mechanism underlying bovine fatty liver. In fact, we found that low-level administration of the recombinant bovine TNFα (rbTNFα) for 7 d promoted liver inflammation and TG accumulation [11]. However, late-lactation animals were used in that work, and it is unclear if this model is applicable to cows in the first week of lactation, when fatty liver naturally occurs. Beyond direct promotion of fatty liver, hepatic inflammation may also affect glucose production. Bradford et al. [11] reported that rbTNFα administration reduced liver gluconeogenic gene expression in late-lactation cows. If inflammatory cytokines impair glucose production in early-lactation animals, the resulting hypoglycemia would likely increase adipose TG mobilization and metabolic disorders.

We hypothesized that metabolic stress in early-lactation cows is exacerbated by inflammatory challenge, thereby adversely affecting production and health. Therefore, the objective of this study was to determine whether exogenous rbTNFα administration promotes inflammation and TG accumulation, impairs gluconeogenesis, alters lipid metabolism, and affects milk production and health status in early-lactation dairy cows. A greater understanding of the pathological impact of inflammatory pathways in these animals may improve our ability to prevent metabolic disorders and increase production.

## Materials and Methods

The Kansas State University Institutional Animal Care and Use Committee approved all experimental procedures.

### Design and Treatments

A recombinant expression vector encoding the secreted form of bovine TNFα (Entrez Protein Accession AAB84086.1, region 77–233) was expressed in *Escherichia coli* and purified by a commercial laboratory (GenScript Corp., Piscataway, NJ). Purity of the isolated rbTNFα was verified by SDS-PAGE, and endotoxin was removed using polymyxin B until contamination was less than<1 EU/µg protein. Thirty-three Holstein cows (9 primiparous and 24 multiparous; body condition score: 3.26±0.31, body weight 741±83 kg; mean ± SD) were randomly assigned to 1 of 3 treatments at parturition. Treatments were balanced within parity, and dystocia did not differ by treatment (calving difficulty scores: 1.36, 1.18, and 1.18±0.5 for Control, 1.5 µg/kg and 3.0 µg/kg TNF, respectively; 1 to 5 scale, with 1 = no difficulty). Treatments (dose per d) were no rbTNFα (Control; 10% glycerol in saline), 1.5 µg rbTNFα/kg body weight in saline with 10% glycerol, and 3.0 µg rbTNFα/kg body weight in saline with 10% glycerol. These doses are slightly less and slightly more, respectively, than the 2.0 µg/kg dose that was previously shown to alter hepatic nutrient metabolism in lactating cows [10]. Glycerol was included in the solution to improve solubility of the rbTNFα protein. Cows were injected subcutaneously once daily (1600 h) for the first 7 d of lactation. Cows were milked 3 times daily (0400, 1200, and 2000 h) in a milking parlor and fed twice daily (0800 and 1600 h) for *ad libitum* intake of a diet formulated to meet National Research Council (NRC) [12] nutrient requirements. Ingredient and nutrient composition of the diet are shown in Table 1.

**Table 1:** Ingredient and nutrient composition of the diet.

**Ingredient,% of dry matter**	
Corn silage	22.2
WCGF^1^	30.3
Alfalfa hay	20.4
Cottonseed	4.9
Corn grain	9.2
Sorghum grain	4.0
Micronutrient premix^2^	9.0
**Nutrient,% of dry matter**	
Dry matter (% as-fed)	58.6
Crude protein	17.3
Acid detergent fiber	17.8
Neutral detergent fiber	31.8
Ether extract	5.0
Ash	9.1
Net energy for lactation^3^ (Mcal/kg dry matter)	1.72

1 Wet corn gluten feed (Sweet Bran; Cargill Inc., Blair, NE).

2 Premix consisted of 54.9% expeller soybean meal, 14.3% limestone, 10.2% sodium bicarbonate, 10.0% calcium salts of long-chain fatty acids (Megalac-R; Arm & Hammer Animal Nutrition, Princeton, NJ), 2.6% Diamond V XP yeast (Diamond V Mills, Inc., Cedar Rapids, IA), 2.0% magnesium oxide, 1.6% potassium carbonate, 1.6% salt, 1.6% Vitamin E premix (44 IU/g), 0.6% 4-Plex (Zinpro Corp., Eden Prairie, MN; consists of zinc 2.58%, manganese 1.48%, copper 0.90%, cobalt 0.18%, methionine 8.21%, and lysine 3.80%), 0.3% selenium premix (0.06% Se), 0.1% Vitamin A premix (30 kIU/g), 0.1% Vitamin D premix (30 kIU/g), 0.1% Rumensin 90 (Elanco Animal Health, Greenfield, IN), and 0.1% Zinpro 100 (Zinpro Corp., Eden Prairie, MN; consists of 10% zinc and 30% methionine).

3 Estimated according to NRC [12].

### Sample and Data Collection and Analysis

#### Feed Intake, Milk Production, Energy Balance, and Health Monitoring

During the 7-d experimental period, intake of feed and water and milk yield were recorded daily. Milk samples were collected from d 4 to 7 of treatment at each of the 3 milkings for analysis of milk components. Samples were analyzed for concentrations of fat, true protein, lactose (B-2000 Infrared Analyzer; Bentley Instruments Inc., Chaska, MN), urea nitrogen (MUN spectrophotometer, Bentley Instruments Inc.), and somatic cells (SCC 500, Bentley Instruments Inc., Heart of America DHIA, Manhattan, KS). Somatic cell linear score was calculated as described by Shook [13]: log_2_(somatic cell count/100) +3. Energy balance was calculated for each cow using the following equation from NRC [12]: energy balance = net energy intake − (net energy of maintenance + net energy of lactation). Net energy intake = dry matter intake × net energy density of the diet [12]; net energy of maintenance = 0.08× body weight^0.75^; net energy of lactation = [(0.0929× fat%)+(0.0547×protein%)+(0.0395× lactose%)] × milk yield. In all equations, energy is expressed in Mcal and mass in kg.

Cows were monitored daily for health status. Ketosis was recorded when urine acetoacetate exceeded 80 mg/dL on any day or 40 mg/dL for 2 consecutive days (Ketostix; Bayer Corp. Diagnostics Division, Elkhart, IN). Fever was diagnosed when a cow had a rectal temperature greater than 39.4°C. Other disorders or diseases, including mastitis, metritis, and milk fever, were diagnosed according to the guidelines by Kelton et al. [14].

#### Plasma Metabolites and Hormones

Blood samples were collected from coccygeal vessels daily (0800 h) in 2 tubes, one containing potassium EDTA and the other containing potassium oxalate with sodium fluoride as a glycolytic inhibitor (Vacutainer; Becton Dickinson, Franklin Lakes, NJ). Blood was centrifuged at 2,000× *g* for 10 min immediately after sample collection, and plasma was frozen at −20°C until further analysis. Plasma samples collected daily during the 7-d treatment period were analyzed for NEFA (NEFA-HR; Wako Chemicals USA Inc., Richmond, VA), glucose (kit #439-90901; Wako Chemicals USA Inc.), BHBA (kit #H7587-58; Pointe Scientific Inc., Canton, MI), and TG (#10010303; Cayman Chemical, Ann Arbor, MI). Plasma samples collected on d 0, 3, 5, and 7 of treatment were analyzed for TNFα (an ELISA method described by Farney et al. [15]), insulin (ELISA kit #10-1201-01; Mercodia AB, Uppsala, Sweden), and haptoglobin (ELISA kit #2410-7; Life Diagnostics, West Chester, PA). Plasma samples collected on d 7 of treatment were analyzed for 3-methylhistidine. Samples were prepared by mixing 500 µL of plasma with 500 µL of Seraprep (Pickering Labs, Mountain View, CA) and frozen overnight. The deproteinized plasma was then thawed, vortexed, and centrifuged at 17,000×*g* for 10 min. The resulting supernatant was analyzed for 3-methylhistidine using Li^+^ cation exchange chromatography and detection by fluorimetry following post column derivitization with o-phthalaldehyde. The HPLC column and all reagents and eluents were purchased from Pickering Labs (Mountain View, CA). Column flow rate was 0.375 mL/min and column temperature was maintained at 36°C. Injection volumn was 10 µL. Eluent 1 (Li357) was run for 5 min then eluent 2 (Li750) for 15 min and eluent 3 (RG003) for 5 min. The column was then re-equilibrated back to eluent 1 for 20 min. Post column derivitization was performed using a post column mixing T at a flow rate of 0.375 mL/min using 950 mL OPA diluent (OD104) containing 0.3 g o- phthalaldehyde, 2.0 g Thiofluor, and 3 mL 30% Brij 35. The fluorimeter excitation was set to 330 nm and emission to 465 nm.

#### Plasma Eicosanoids

Plasma samples collected on d 0, 1, 3, and 5 of treatment were analyzed for eicosanoids as previously described [16]. Plasma samples (500 µL) were mixed with 1 mL ice cold methanol, 6 µL antioxidant/reducing agent containing EDTA, butylhydroxytoluene, triphenylphosphine, and indomethacin (4 µL/mL), 200 µL of a mixture of internal standards, and 1 µL of formic acid. Sample mixtures were centrifuged at 4°C for 10 min at 3,000×*g*, and the supernatant was used for solid-phase extraction using Strata-X SPE columns (Phenomenex Inc., Torrance, CA). Eicosanoids were isolated using 2 distinct ultra-high–pressure liquid chromatography (UPLC) and mass spectrometry (UPLC-MS) methods. Both methods used reverse-phase liquid chromatography on an Acquity UPLC BEH C18 1.7-µm column (2.1×100 mm; Waters Corp., Milford, MA) at a flow rate of 0.6 mL/min at 35°C and a single-quadrupole H-class Acquity SQD mass spectrometer in electrospray negative ionization mode (Waters Corp.). The electrospray voltage was −3 kV, and the turbo ion spray source temperature was 450°C. Nitrogen was used as the drying agent. For each method, a 10-µL sample was injected 3 consecutive times using a 10-µL injection loop. An isocratic mobile phase consisting of acetonitrile/water/formic acid (45/55/0.01; vol/vol/vol) with an analysis time of 15 min was used to measure leukotriene B_4_ (LTB_4_), thromboxane B_2_ (TXB_2_), prostaglandin E_2_ (PGE_2_), prostaglandin F_2_ (PGF_2_), lipoxin A_4_, resolvin D_1_, and resolvin D_2_. The second method used an isocratic mobile phase of acetonitrile/methanol/water/formic acid (47.4/15.8/26.8/0.01; vol/vol/vol/vol) and an analysis time of 10 min to analyze 9-hydroxyoctadecadienoic acid (HODE), 13-HODE, 9-oxo-octadecadienoic acid (oxoODE), 13-oxoODE, 5-hydroxyeicosatetraenoic acid (HETE), 15-HETE, 7-maresin 1, leukotriene D_4_ (LTD_4_), and protectin.

Quantitation of eicosanoid concentrations was performed with Waters Empower 2 software (Waters Corp.). A linear calibration curve with 5 points (R^2^>0.99) was generated for each eicosanoid with standards and internal standards purchased from Cayman Chemical Co. (Ann Arbor, MI). The curves ranged from 0.0024 to 2.38 ng/µL. Empower 2 identified the sample peak by matching its retention time with the standard. A response was calculated for each matched peak by dividing the sample peak's response by its internal standard's response. This response was multiplied by the concentration of the internal standard for each analyte. The concentration of each analyte was calculated using the response peak and injection volume.

#### Glucose Turnover Rate

After daily blood sampling on d 5 of treatment, jugular vein catheters were placed and at least 18 h of recovery were allowed prior to sample collection through the catheters. On treatment d 7, cows were given a glucose bolus containing U-^13^C-glucose (99 atom%, Sigma-Aldrich Co., St. Louis, MO) through jugular catheters [17]. Jugular blood samples were collected 10 min prior to infusion and at 10, 20, 30, 40, 50, 60, 90, and 120 min post-infusion. Each cow received approximately 1 g of U-^13^C-glucose in 50 mL of sterile saline; syringes were weighed immediately before and after infusion to determine the exact amount administered. Catheters were flushed with a sterile solution of 3.5% sodium citrate after the labeled glucose bolus (20 mL) and after each blood sample collection (5 mL). Plasma glucose concentration was quantified to verify that steady-state conditions were met.

Plasma samples collected for the glucose turnover assay were analyzed for U-^13^C-glucose enrichment (Metabolic Solutions, Inc., Nashua, NH). Glucose was extracted and converted to aldonitrile pentaacetate derivative [18], and negative chemical ionization GC/MS (Hewlett-Packard 5890; Agilent Technologies, Santa Clara, CA) was used to analyze derivatized samples. The isotopic composition of the glucose was determined by monitoring unlabeled (M+0: m/z = 328) versus U-^13^C-labeled (M+6: m/z = 334) glucose derivatives. This approach, as opposed to oxidation of glucose and measurement of CO_2_ enrichment, ensures that results are not biased by carbon recycling via the Cori cycle. Turnover rate of plasma glucose was calculated from the disappearance curve for U-^13^C-glucose. Enrichment of plasma glucose for each animal was fitted to an exponential decay curve according to the following equation: E_t_ = E_0_× e^−kt^, where t = time relative to infusion (min), E_t_ = enrichment of plasma glucose (U-^13^C-glucose: unlabeled glucose ratio) at time t, E_0_ = enrichment at time t = 0, and k = rate constant (min^−1^). After using the best-fit equations to determine k and E_0_, the total glucose pool was calculated by the following equation: G = M÷E_0_, where G = total glucose pool (g) and M = mass of tracer infused (g). Plasma glucose turnover rate (GTR, g/min) was calculated according to the equation GTR = G×k. Samples collected 10 min prior to infusion of U-^13^C-glucose were also analyzed to verify the lack of natural occurrence of the M+6 isotopomer.

#### Liver TG

At the end of treatment d 7, liver samples were collected by percutaneous biopsy as described by Morey et al. [19]. For analysis of TG content, approximately 20 mg of liver was placed into 500 µL of chilled phosphate buffered saline (pH 7.4) and homogenized. The homogenate was centrifuged at 2,000× *g* for 10 min at 4°C, and 100 µL of the supernatant was then removed for free glycerol and total protein analyses. Triglyceride content was measured using a method adapted from Starke et al. [20]. The remaining liver homogenate was incubated with 100 µL of lipase (porcine pancreatic lipase; MP Biomedicals, Solon, OH) for 16 h at 37°C, and glycerol content was then determined by an enzymatic glycerol phosphate oxidase method (#F6428, Sigma-Aldrich Co.). Triglyceride content was calculated based on the difference between glycerol concentrations before and after lipase digestion. Total protein content of the original homogenate was analyzed by a Coomassie blue [21] colorimetric method (kit #23236; Thermo Scientific Pierce, Rockford, IL). To avoid potential bias introduced by differences in moisture content of liver samples, liver TG concentration was normalized by protein concentration, which is unaltered in fatty liver [22].

#### Transcript Abundance

Total RNA was extracted from liver tissue using a commercial kit (RNeasy Lipid Tissue Mini Kit; Qiagen Inc., Valencia, CA) according to the manufacturer's instructions. Two micrograms of total RNA was used as the template for the reverse transcriptase reaction using random primers (High-Capacity cDNA RT Kit; Applied Biosystems, Foster City, CA). Quantitative real-time PCR was performed in duplicate on 96-well plates with 5% of the cDNA product in the presence of 200 nM gene-specific forward and reverse primers with real-time SYBR green fluorescent detection using SYBR Green Premix reagent (7500 Fast Real-Time PCR System, Applied Biosystems). Primers were designed (www.ncbi.nlm.nih.gov/tools/primer-blast/) using GenBank sequences (Table 2). Data were recorded and analyzed with Sequence Detector software (Applied Biosystems). All sample values were normalized against ribosomal protein subunit 9 (*RPS9*) values [23], and relative gene expression was quantified by using the 2^−ΔCt^ method. Treatment did not influence the Ct value for *RPS9* (*P* = 0.56), suggesting that it served as a valid control gene.

**Table 2:** Primers used for real-time PCR gene expression analysis.

Gene^1^	Sequences of primers (5′ to 3′)	Accession number^2^	Amplicon region	Median Ct	Efficiency^3^
*AGPAT1*	Forward, GGAGTCATCTTCATTGACCGGA	NM_177518.1	737–847	29.3	99%
	Reverse, GCCCTCAGGAAAAACCCA				
*ApoB*	Forward, TCCTTGATTCCACATGCAGCT	XM_003582812.1	11567–11666	20.6	94%
	Reverse, GGTGTGCAAAGGATGCGTTAG				
*CPT1a*	Forward, CTTCCCATTCCGCACTTTC	XM_002699420.2	1950–2033	22.6	93%
	Reverse, CCATGTCCTTGTAATGAGCCA				
*PC*	Forward, CTTCAAGGACTTCACTGCCACC	NM_177946.4	3160–3276	21.4	98%
	Reverse, GCCAAGGCTTTGATGTGCA				
*PCK1*	Forward, CGAGAGCAAAGAGATACGGTGC	NM_174737.2	427–542	18.5	98%
	Reverse, TGACATACATGGTGCGACCCT				
*RPS9*	Forward, GAACAAACGTGAGGTCTGGAGG	NM_001101152.2	170–261	19.2	92%
	Reverse, TTACCTTCGAACAGACGCCG				
*TNFα*	Forward, AAGTAACAAGCCGGTAGCCCA	NM_173966.3	448–538	27.9	103%
	Reverse, CTTCCAGCTTCACACCGTTG				

1 *AGPAT1*: 1-acylglycerol-3-phosphate O-acyltransferase 1; *ApoB*: apolipoprotein B; *CPT1a*: mitochondrial carnitine palmitoyltransferase 1A; *PC*: pyruvate carboxylase; *PCK1*: phosphoenolpyruvate carboxykinase 1; *RPS9*: ribosomal protein S 9; *TNFα*: tumor necrosis factor α.

2 From NCBI Entrez Nucleotide Database (http://www.ncbi.nlm.nih.gov/sites/entrez?db=nucleotide).

3 The coefficients of determination (R^2^) for all genes were greater than 0.98.

#### Western Blot

Relative protein abundance of IκBα, c-Jun, phosphorylated c-Jun, and TNFα in liver samples was determined by Western blot. Liver samples (∼20 mg) were homogenized at 4°C in RIPA lysis buffer containing a broad-spectrum protease inhibitor cocktail (Protease Inhibitor Cocktail I; EMD Millipore, Billerica, MA) and a phosphatase inhibitor (PhosphoStop; Santa Cruz Biotechnology, Santa Cruz, CA). The homogenate was centrifuged at 15,000× *g* for 10 min at 4°C, and total protein concentration of the supernatant was measured [21]. Forty micrograms of total protein from liver tissue was heated at 90°C for 5 min, vortexed, and loaded onto a 4 to 12% Tris-HCl gel for electrophoresis. Samples were separated by SDS-PAGE and dry-transferred onto nitrocellulose membranes (iBlot; Invitrogen, Carlsbad, CA). Membranes were blocked for 2 h in blocking buffer (5% dry milk in Tris-HCl buffer, pH 7.5, with 0.05% Tween 20). After incubation with blocking buffer, the membranes were washed 3 times for 5 min each with washing buffer (phosphate-buffered saline, pH 7.5, containing 0.05% Tween 20). Membranes were then incubated with primary antibodies (diluted 1∶250) from Santa Cruz Biotechnology against IκBα (catalog #sc-847), c-Jun (#sc-44), and phosphorylated c-Jun (#sc-822) overnight at 4°C. After washing, secondary antibody diluted 1∶10,000 was incubated for 1 h at room temperature. Antibody incubation and detection for TNFα was carried out as previously described [11]. Immunodetection was performed by chemiluminescence (West-Dura; Thermo Scientific, Waltham, MA), and bands were quantified by scanning densitometry (ChemiDoc-It Imaging System; UVP Inc., Upland, CA).

### Calculations and Statistical Analyses

One cow that received 3 µg/kg rbTNFα developed a severe fever on d 6 of treatment, and the d 7 treatment was not given. Consequently, the liver biopsy and glucose turnover test were not conducted for this cow, but all other data were retained through d 6. Plasma glucose data indicated that 2 cows did not meet steady-state conditions during the glucose turnover test; these cows were not used for turnover rate analysis. In total, data from 33, 32, and 30 cows were used for production responses and plasma analyses, liver tissue analyses, and glucose turnover analysis, respectively. Data were analyzed using the MIXED Procedure of SAS (version 9.2; SAS Institute Inc., Cary, NC) to assess the fixed effects of treatment, parity, time, and 2- and 3-way interactions; cow was included as a random effect. Data were log-transformed for analysis when necessary (including plasma concentrations of TNFα and 3-methylhistidine, and hepatic transcript abundance of *AGPAT1*, *PCK1*, and *CPT1a*) to achieve a normal distribution of residuals, and data presented are back-transformed in these cases. Repeated measures over time were modeled with either autoregressive or heterogeneous autoregressive covariance structures, depending on which analysis had the lowest Bayesian Information Criterion value. Denominator degrees of freedom were estimated using the Kenward-Rogers method. Values were deemed outliers and omitted from analysis when Studentized residuals were>3.5 or<−3.5. Interactions were investigated when *P*<0.10 using the slice option, and slices were declared significant at *P*<0.05. Contrasts were used to evaluate Control vs. rbTNFα treatments and 1.5 µg/kg vs. 3.0 µg/kg rbTNFα doses. Health disorders were analyzed using JMP (version 8.0; SAS Institute Inc., Cary, NC). A nominal logistic analysis was first run for each health disorder to assess the fixed effects of treatment, parity, and treatment × parity interaction. For those disorders with an interaction *P*<0.10, Fisher's exact test was used to assess rbTNFα and dose contrasts within each parity group. For all other disorders, Fisher's exact test was used to test these contrasts across all animals. Significance was declared at *P*≤0.05 and tendencies at 0.05<*P*<0.10.

## Results

### Inflammatory Signals

**Figure 1:**
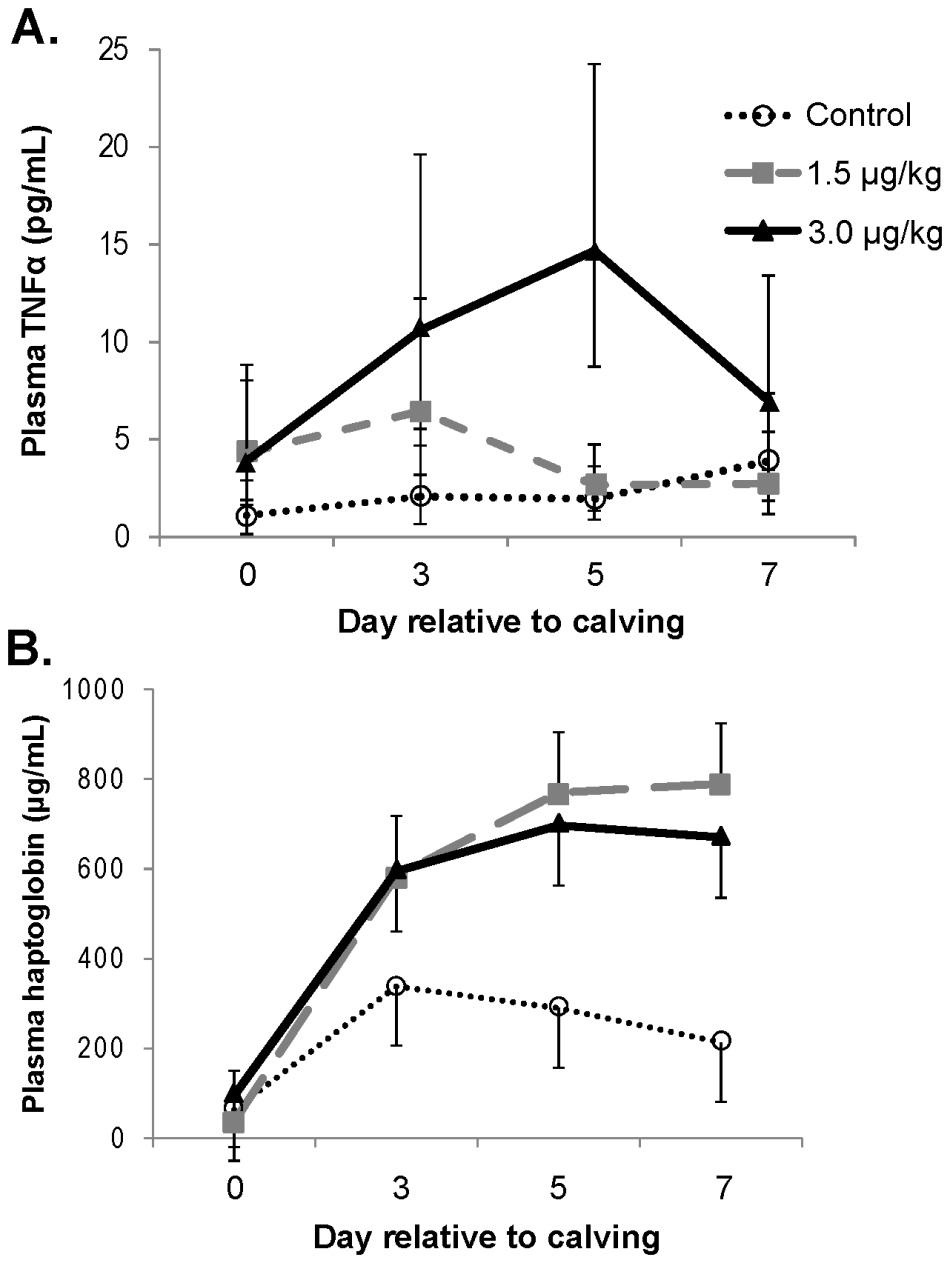
Plasma concentrations of TNFα and haptoglobin during 7 days of rbTNFα or Control administration. (A) Plasma TNFα tended to be increased by rbTNFα treatments (*P* = 0.09), but no difference was detected between 1.5 and 3.0 µg/kg rbTNFα treatments (*P* = 0.19). (B) Haptoglobin differed between rbTNFα treatments and Control (*P* = 0.01), but not between 1.5 and 3.0 µg/kg rbTNFα treatments (*P* = 0.68). Values are least squares means ± SEM, n = 10–11.

Compared with Control, plasma TNFα concentrations tended to be increased (*P* = 0.09) by rbTNFα treatments, but 1.5 µg/kg did not differ (*P* = 0.19) from 3 µg/kg rbTNF (Figure 1A). Plasma haptoglobin concentrations were greater (*P*<0.01, Figure 1B) in rbTNFα treatments than Control, but did not differ between 1.5 and 3 µg/kg rbTNF (*P* = 0.68). Across the 3 groups, haptoglobin levels were comparable (*P* = 0.94) at parturition, but increased during the first week of lactation in response to rbTNFα. On d 5 and 7 of treatment, rbTNFα treatments increased haptoglobin concentration by ∼2.5- and ∼3.5-fold, respectively, compared with Control.

**Figure 2:**
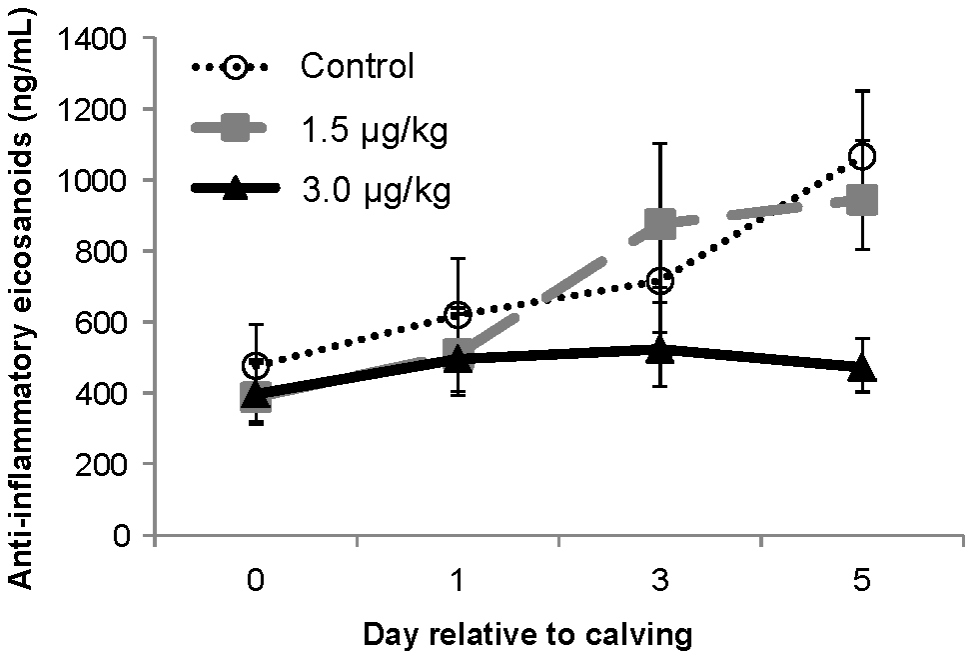
Composite plasma concentration of anti-inflammatory eicosanoids during the first 5 days of rbTNFα or Control administration. The total anti-inflammatory eicosanoid concentration represents the sum of resolvin D_1_ and D_2_, protectin, lipoxin A_4_, 7-maresin 1, 9-oxoODE, and 13-oxoODE concentrations [27]. The 3.0 µg/kg dose tended to differ from the 1.5 µg/kg rbTNFα dose (*P* = 0.08), but no overall rbTNFα was detected (*P* = 0.22). A tendency for a dose by time interaction was also observed (*P* = 0.06), with a significant dose contrast on day 5 (*P*<0.01). Values are least squares means ± SEM, n = 10–11.

Plasma eicosanoid results are shown in Table 3. Most eicosanoids were unaffected (*P*>0.10) by treatments; out of the 16 measured eicosanoids, only 9-oxoODE was decreased (*P* = 0.01) by rbTNFα administration. The pro-inflammatory class as a composite was unaffected by treatments; however, the anti-inflammatory class tended (*P* = 0.08) to be decreased by the 3 µg/kg compared with 1.5 µg/kg rbTNFα dose (Figure 2), though rbTNFα treatment did not differ from Control (*P* = 0.22).

**Table 3:** Concentrations of plasma eicosanoids in early-lactation dairy cows during the experimental period.

	Treatments^2^				*P*-value^3^	
Eicosanoids^1^, ng/mL	Control	1.5 µg/kg	3.0 µg/kg	SEM	C vs. T^4^	Dose^5^
PGE_2_	1.07	1.26	1.21	0.14	0.16	0.88
PGF_2_	1.55	1.61	1.56	0.075	0.36	0.61
LTB_4_ *	0.43	0.43	0.28	0.101	0.87	0.49
LTD_4_	335	305	275	120	0.68	0.74
TXB_2_	33.2	25.5	36.9	6.6	0.86	0.31
9-HODE	30.4	31.7	27.4	3.6	0.89	0.64
13-HODE	28.5	28.7	26.7	3.5	0.60	0.48
5-HETE	94.9	96.1	65.3	18.2	0.26	0.25
15-HETE	4.49	4.50	4.75	0.69	0.64	0.73
Pro-inflammatory class^6^	482	426	384	95	0.40	0.63
Resolvin D_1_	0.172	0.294	0.241	0.053	0.14	0.59
Resolvin D_2_	19.3	16.6	8.6	4.7	0.22	0.26
Lipoxin A_4_	1.84	1.38	1.29	0.33	0.31	0.90
7-maresin 1	472	430	302	69	0.22	<0.10
Protectin	89.9	83.6	49.2	18.7	0.30	0.06
9-oxoODE	54.1	46.0	46.0	3.2	0.01	0.86
13-oxoODE	26.4	27.1	22.5	2.8	0.34	0.43
Anti-inflammatory class^7^	689	636	469	96	0.22	0.08

1 LT: leukotriene; PG: prostaglandin; TX: thromboxane; HODE: hydroxyoctadecadienoic acid; HETE: hydroxyeicosatetraenoic acid; oxoODE: octadecadienoic acid.

2 Treatments: cows were given 0, 1.5, or 3.0 µg/kg body weight rbTNFα injections daily for the first 7 days of lactation, respectively.

3 No treatment by day interactions were significant except as noted.

4 Contrast between Control and rbTNFα treatments.

5 Contrast between 1.5 and 3.0 µg/kg rbTNFα treatments.

6 Pro-inflammatory class is the sum all of PGE_2_, PGF_2_, LTB_4_, LTD_4_, TXB_2_, 9-HODE, 13-HODE, 5-HETE, and 15-HETE concentrations [27].

7 Anti-inflammatory class is the sum of resolvin D_1_ and D_2_, protectin, lipoxin A_4_, 7-maresin 1, 9-oxoODE, and 13-oxoODE concentrations [27].

* A significant treatment × time interaction was detected (*P*<0.04), but treatment contrasts were not significant on any individual day.

Eicosanoids were measured on day 0, 1, 3, and 5 of treatment. Values are least squares means ± SEM, n = 10–11.

Plasma eicosanoids with significant (*P*<0.05) day effects are presented in Table 4. Plasma concentrations of TXB_2_, 15-HETE, and 9-HODE were elevated around parturition and decreased during the first week of lactation. In contrast, resolvin D_2_ and 7-maresin 1 increased gradually after parturition. The pro-inflammatory class decreased (*P* = 0.02) during the first week of lactation, whereas the anti-inflammatory class (*P*<0.01) increased after parturition.

**Table 4:** Concentrations (across treatments) of plasma eicosanoids in early-lactation dairy cows with significant (*P*<0.05) day effects during the experimental period.

	Day^2^					
Eicosanoids^1^, ng/mL	0	1	3	5	SEM	*P*-value
PGF_2_	1.67	1.44	1.41	1.78	0.11	0.02
TXB_2_	52	16	26	35	10	<0.001
Resolvin D_2_	5.1	12.2	21.8	20.4	3.7	<0.01
9-HODE	34.4	29.0	25.8	30.1	3.67	0.04
15-HETE	6.7	4.4	3.7	4.2	1.04	0.03
7-maresin 1	248	358	481	568	59	<0.01
Pro-inflammatory class^3^	531	369	374	460	70	0.02
Anti-inflammatory class^4^	418	538	689	781	85	<0.01

1 PG: prostaglandin; TX: thromboxane; HODE: hydroxyoctadecadienoic acid; HETE: hydroxyeicosatetraenoic acid.

2 Day of lactation. Plasma eicosanoids with significant (*P*<0.05) day effects are presented.

3 Pro-inflammatory class is the sum of PGE_2_, PGF_2_, LTB_4_, LTD_4_, TXB_2_, 9-HODE, 13-HODE, 5-HETE, and 15-HETE concentrations [27].

4 Anti-inflammatory class is the sum of resolvin D_1_ and D_2_, protectin, lipoxin A_4_, 7-maresin 1, 9-oxoODE, and 13-oxoODE concentrations [27].

Values are least squares means ± SEM, n = 31–33.

**Figure 3:**
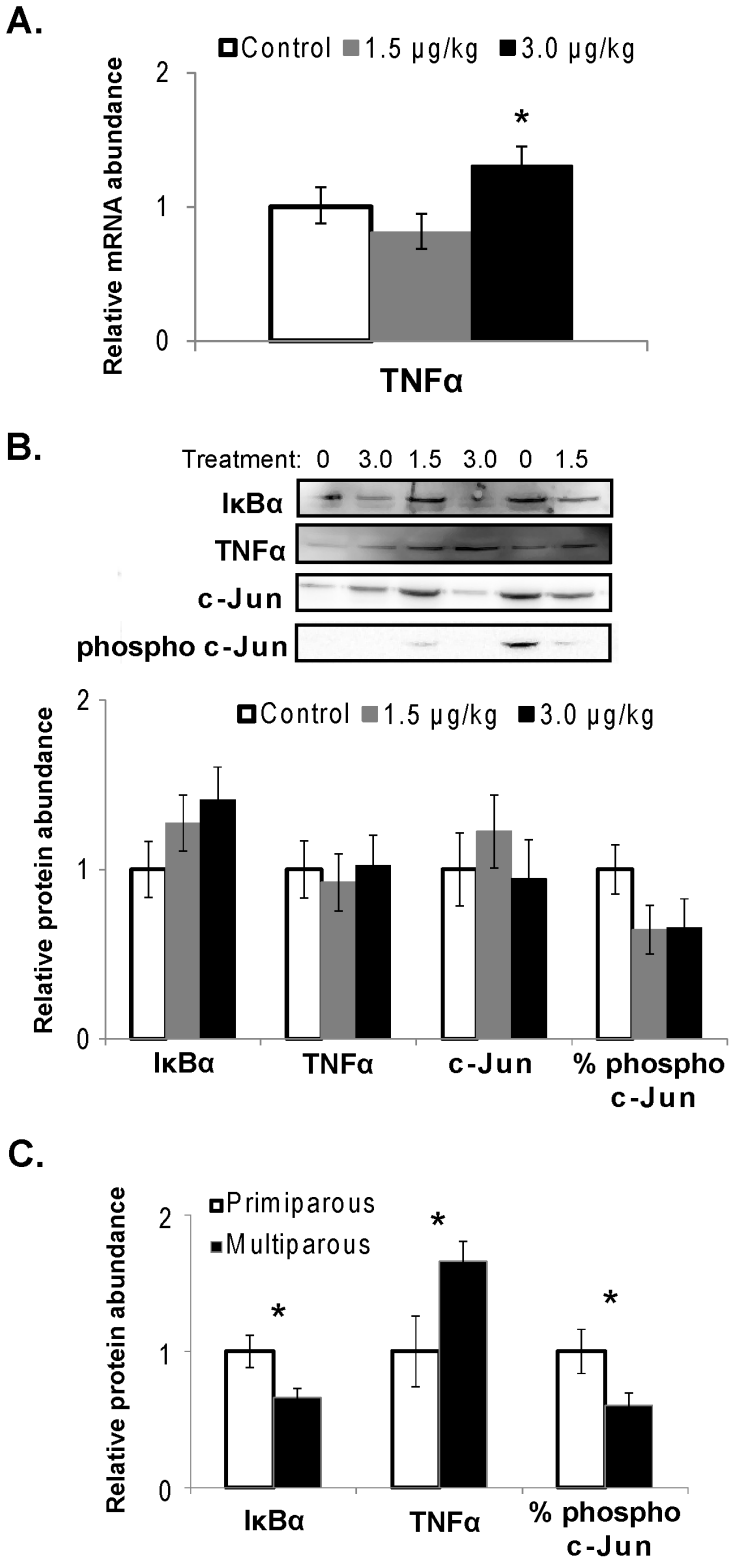
Hepatic mRNA abundance of *TNFα* and protein abundance of key mediators involved in inflammatory pathways. Liver samples were collected after 7 days of rbTNFα or Control administration. (A) Hepatic *TNFα* transcript abundance was increased by 3.0 vs. 1.5 µg/kg rbTNFα treatments (*P* = 0.02), but did not differ between rbTNFα treatments and Control (*P* = 0.73). (B) Western blot images are shown for 6 cows along with densitometry data from analysis of all samples for hepatic IκBα (37 kDa), TNFα (17 kDa), and total and phosphorylated c-Jun (39 kDa). There was a tendency for treatment by parity interaction (*P* = 0.07) for IκBα, reflecting increased (*P* = 0.04) IκBα abundance by rbTNFα treatment in primiparous cows (data not shown). No treatment effects (*P*>0.10) were observed for hepatic TNFα, c-Jun, or relative c-Jun phosphorylation. (C): Parity significantly affected hepatic IκBα (*P* = 0.02), TNFα (*P* = 0.04), and relative c-Jun phosphorylation (*P* = 0.04). Values are means ± SEM, n = 10–11 (A and B) or 9–24 (C).

As shown in Figure 3A, hepatic transcript abundance of *TNFα* was increased (*P* = 0.02) by 3 µg/kg compared with 1.5 µg/kg rbTNFα, but overall, rbTNFα treatments did not differ from Control (*P* = 0.73). There were no treatment effects (*P*>0.10) for protein abundance of TNFα, c-Jun, or relative c-Jun phosphorylation (Figure 3B). There was a tendency for a treatment × parity interaction (*P* = 0.07) for IκBα, reflecting increased (*P* = 0.04) IκBα abundance by rbTNFα treatment in primiparous cows (data not shown). Compared with primiparous cows, IκBα was decreased (*P* = 0.02), TNFα was increased (*P* = 0.04), and relative c-Jun phosphorylation was decreased (*P* = 0.04) in multiparous cows (Figure 3C).

### Energetics

Dry matter and water intake were decreased (*P*<0.05) by rbTNFα treatments, but no dose effect (*P*>0.10) was observed (Table 5). Cows were in negative energy balance during the experimental period, and the energy balance values were not affected (*P*>0.10) by treatments. Milk yield was decreased (*P* = 0.03) by rbTNFα treatments, with no dose effect (*P* = 0.76). Similar to milk yield, milk energy output was decreased (*P*<0.01) by rbTNFα administration. Milk fat percentage was decreased (*P* = 0.02) by 3.0 µg/kg rbTNFα compared with the 1.5 µg/kg dose, but overall, rbTNFα treatments did not differ from Control (*P* = 0.55). Nevertheless, milk fat yield was decreased (*P* = 0.01) in cows treated with rbTNFα, and there was a tendency for decreased (*P* = 0.07) milk fat yield for the 3.0 µg/kg rbTNF dose compared with 1.5 µg/kg. Milk protein and lactose content were unaffected by treatment (*P*>0.10), but yields of both were decreased (*P*<0.01) by rbTNFα administration. Milk urea nitrogen concentration and somatic cell linear score were not affected (*P*>0.10) by treatments.

**Table 5:** Production responses in early-lactation dairy cows during the experimental period.

	Treatments^1^				*P*-value^2^	
Item	Control	1.5 µg/kg	3.0 µg/kg	SEM	C vs. T^3^	Dose^4^
Dry matter intake, kg/d	13.7	12.1	10.4	0.80	0.02	0.15
Water intake, L/d	79.8	71. 9	66.5	3.9	0.04	0.34
Milk yield, kg/d	33.7	29.1	28. 4	1.7	0.03	0.76
Milk fat,%	5.41	6.00	5.18	0.24	0.55	0.02
Milk fat, kg/d	2.08	1.87	1.59	0.11	0.01	0.07
Milk protein,%	3.51	3.57	3.37	0.11	0.75	0.19
Milk protein, kg/d	1.29	1.11	1.01	0.05	<0.01	0.19
Milk lactose*,%	4.52	4.52	4.45	0.05	0.65	0.33
Milk lactose, kg/d	1.65	1.39	1.32	0.08	<0.01	0.55
Milk energy output, Mcal/d	36.8	32.1	28.7	1.6	<0.01	0.13
Energy balance, Mcal/d	−23.6	−21.6	−22.0	1.6	0.36	0.89
Milk urea nitrogen, mg/dL	11. 1	12.7	12.3	0.75	0.13	0.75
Somatic cell linear score	3.27	2.49	3.13	0.51	0.50	0.39

1 Treatments: cows were given 0, 1.5, or 3.0 µg/kg body weight rbTNFα injections daily for the first 7 days of lactation, respectively.

2 No treatment by day interactions were significant except as noted.

3 Contrast between Control and rbTNFα treatments.

4 Contrast between 1.5 and 3.0 µg/kg rbTNFα treatments.

* There was a treatment by day interaction (*P* = 0.04) for milk lactose%.

Values are least squares means ± SEM, n = 10–11.

### Metabolism

Plasma concentrations of glucose, insulin, BHBA, NEFA, TG, and 3-methylhistidine, and liver TG were not affected (*P*>0.10, Table 6) by rbTNFα treatment. Glucose turnover rate (Table 6) was unaffected (*P = *0.18) by rbTNFα treatments. When expressed relative to dry matter intake (daily glucose turnover rate/day 7 dry matter intake), 3 µg/kg rbTNFα was greater (*P<*0.01) than other treatments.

**Table 6:** Plasma metabolites and hormones, liver triglycerides, and glucose turnover rate in early-lactation dairy cows.

	Treatments^1^				*P*-value^2^	
Item	Control	1.5 µg/kg	3.0 µg/kg	SEM	C vs. T^3^	Dose^4^
Plasma BHBA, µM	996	1108	1186	154	0.40	0.72
Plasma glucose, mg/dL	50.8	51.9	51.7	2.l	0.68	0.94
Plasma insulin, ng/mL	0.28	0.31	0.32	0.07	0.35	0.74
Plasma NEFA, µM	857	854	757	83	0.62	0.41
Plasma TG, mg/dL	13.5	12.9	12.4	1.1	0.52	0.73
Plasma 3-methylhistidine, µM	10.2	10.0	12.2	1.7	0.58	0.28
Liver TG, mg/g protein	769	750	702	106	0.34	0.58
Glucose turnover rate, g/min	3.73	3.04	3.23	0.35	0.18	0.73
Glucose turnover rate, g/kg dry matter intake	389	335	480	29	0.32	<0.01

1 Treatments: cows were given 0, 1.5, or 3.0 µg/kg body weight rbTNFα injections daily for the first 7 days of lactation, respectively.

2 No treatment by day interactions were significant.

3 Contrast between Control and rbTNFα treatments.

4 Contrast between 1.5 and 3.0 µg/kg rbTNFα treatments.

Values are least squares means ± SEM, n = 10–11.

**Figure 4:**
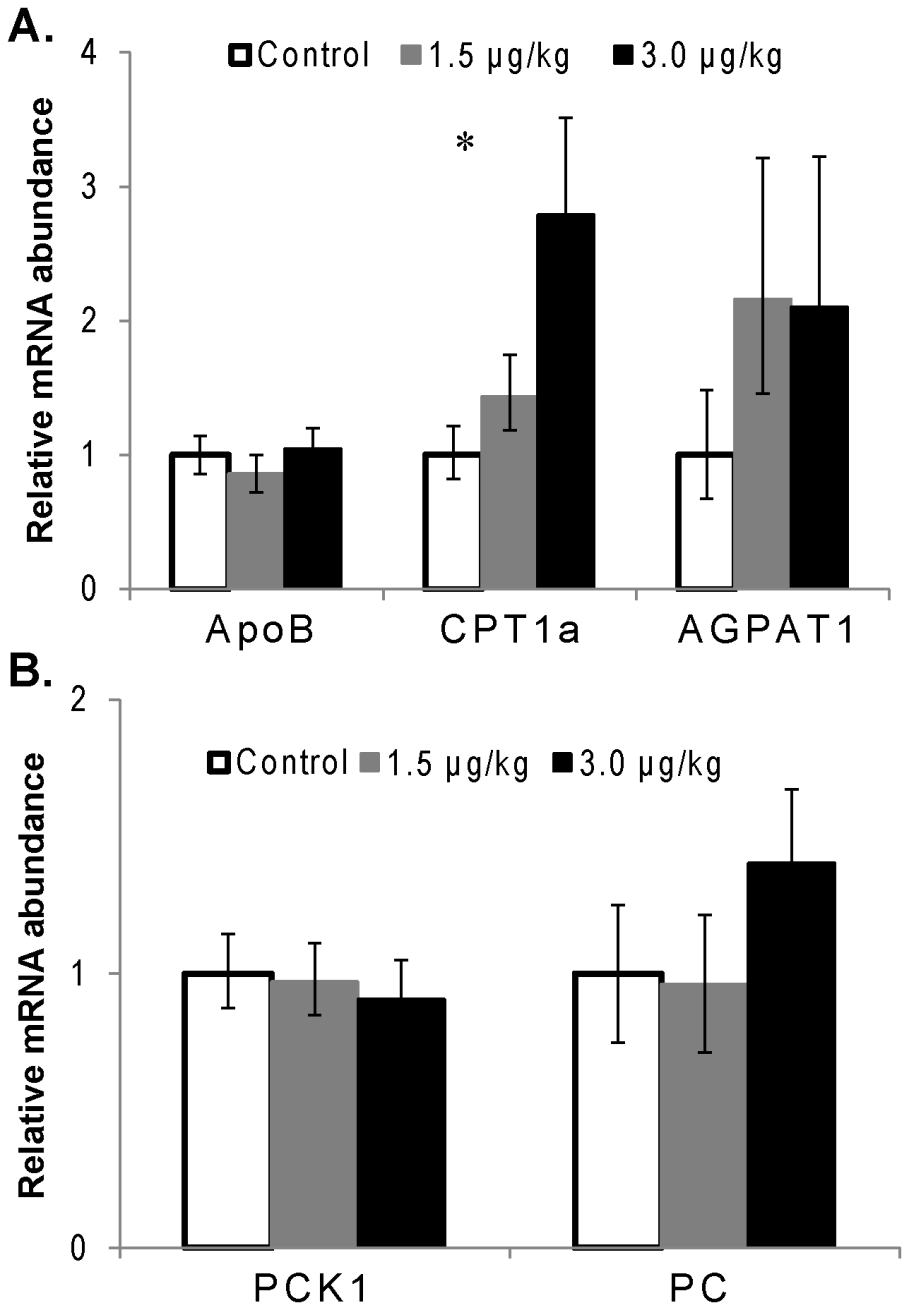
Hepatic abundance of transcripts involved in lipid metabolism (A) and gluconeogenesis (B). Liver samples were collected after 7 days of rbTNFα or Control administration. Differences were observed between rbTNFα treatments and Control (*P*<0.01), and between 1.5 and 3.0 µg/kg rbTNFα treatments (*P* = 0.04) for *CPT1a*. No treatment effects (*P*>0.10) were detected for *ApoB*, *AGPAT1*, *PCK1*, or *PC*. *AGPAT1*: 1-acylglycerol-3-phosphate O-acyltransferase 1; *ApoB*: apolipoprotein B; *CPT1a*: mitochondrial carnitine palmitoyltransferase 1A; *PC*: pyruvate carboxylase; *PCK1*: phosphoenolpyruvate carboxykinase 1. Values are means ± SEM, n = 10–11.

As shown in Figure 4, the mRNA abundance of mitochondrial carnitine palmitoyltransferase 1a (*CPT1a*) was increased (*P*<0.01) by rbTNFα treatments, with 3 µg/kg greater (*P* = 0.04) than 1.5 µg/kg rbTNF. No treatment effects (*P*>0.10) were detected for apolipoprotein B (*ApoB*), 1-acylglycerol-3-phosphate O-acyltransferase 1 (*AGPAT1*), phosphoenolpyruvate carboxykinase 1 (*PCK1*), or pyruvate carboxylase (*PC*).

### Health Disorders

Incidence of health disorders is shown in Table 7. There was a tendency for increased (*P* = 0.08) risk of experiencing 1 or more health disorders for 3 µg/kg compared with 1.5 µg/kg rbTNFα, although the overall rbTNFα effect was not significant (*P* = 0.25). Ketosis incidence reported in Table 7 was based on detection of urine ketones; of the cows identified by this method, 5 cows (1 control, 2 on each of the rbTNFα treatments) had plasma BHBA concentrations that exceeded 3 mM on at least one day, identifying them as clinical cases, whereas the 2 others exceeded the 1.4 mM threshold for subclinical ketosis [3].

**Table 7:** Health disorders in early-lactation dairy cows during the experimental period.

	Treatments^1^			
Item^2^	Control	1.5 µg/kg	3.0 µg/kg	Total
Ketosis	1	3	3	7
Subclinical mastitis	1	0	2	3
Respiratory distress	0	0	2	2
Metritis	1	0	0	1
Milk fever	0	0	1	1
Fever	0	0	2	2
≥1 event (fever excluded)	2	3	7	12

1 Treatments: cows were given 0, 1.5, or 3.0 µg/kg body weight rbTNFα injections daily for the first 7 days of lactation, respectively. There were 11 cows at risk in each treatment.

2 Ketosis was recorded when the urine ketone dipstick test (Ketostix; Bayer Corp. Diagnostics Division, Elkhart, IN) detected acetoacetate>80 mg/dL on any day or>40 mg/dL for 2 consecutive days. Fever designates that a cow had a rectal temperature greater than 39.4°C. Other health disorders were diagnosed according to the guidelines by Kelton et al. [14]. Fever was excluded from the summary data because of the possibility that it was a direct response to treatment rather than a sign of infection.

## Discussion

Our previous research demonstrated that administration of 2 µg/kg body weight rbTNFα daily for 7 d was adequate to mimic a chronic low-grade inflammatory state without causing acute systemic inflammation in late-lactation cows [11]. Therefore, in the present study, we administered 1.5 and 3 µg/kg rbTNFα in an attempt to characterize a similar low-grade inflammation in early-lactation cows, which experience dramatic energy demands of milk production and tremendous metabolic stress compared with late-lactation animals. Although blood samples were collected 16 h after the previous injection, we still observed a tendency for elevated plasma TNFα concentrations in treated cows, indicating a sustained chronic inflammation status in cows receiving rbTNFα. Moreover, 3 µg/kg rbTNFα administration increased hepatic transcript abundance of *TNFα*, further supporting that treatment enhanced pro-inflammatory responses.

We also evaluated plasma concentrations of haptoglobin and eicosanoids, and the protein abundance of key inflammatory mediators in the liver. Haptoglobin is an acute phase protein primarily released by the liver in inflammation [24]. Our finding that rbTNFα increased haptoglobin agrees with a previous study that daily administration of 2.5 µg/kg rbTNFα for 7 d dramatically elevated plasma haptoglobin in lactating cows [25]. Interestingly, haptoglobin elevation has been associated with bovine fatty liver [4], suggesting that liver TG accumulation may be associated with inflammation. However, recent research indicated that excess NEFA influx to the liver, rather than hepatic TG accumulation *per se*, causes liver damage through promoting lipotoxicity, oxidative stress, and inflammatory reactions [26]. In the present study, neither NEFA nor liver TG were affected by treatments, suggesting that rbTNFα increased haptoglobin independent of altered lipid mobilization.

Eicosanoids are a family of signaling molecules produced by oxidation of 20-carbon essential fatty acids. They exert complex control over inflammation, and can either enhance (such as certain types of prostaglandins, thromboxanes, and leukotrienes) or resolve (such as resolvins, protectins, and maresins) inflammatory responses depending on their types and the timing of production [27]. The increased lipolysis and elevated plasma NEFA in early lactation may increase the supply of fatty acid substrates for eicosanoid production, thereby affecting the duration and magnitude of inflammation. In the present study, 6 out of the 16 measured plasma eicosanoids were significantly altered over time, but few treatment effects were detected for eicosanoids. One interesting finding was that the highest dose of rbTNFα tended to suppress the anti-inflammatory eicosanoid class, primarily reflecting decreased concentrations of 7-maresin 1, protectin, and 9-oxoODE. We are unaware of evidence in the literature of cytokines inhibiting release of anti-inflammatory eicosanoids, but such an effect is consistent with the pro-inflammatory effects of TNFα [7]. We cannot rule out the possibility that decreases in anti-inflammatory eicosanoids may have been driven by an unknown mediator rather than a direct effect of TNFα. Still, it is intriguing that rbTNFα administration apparently did more to suppress the production of resolving lipid mediators than to enhance the production of inflammatory lipids, and this result should inform choices about measures of interest in future studies.

We found that concentrations of pro-inflammatory eicosanoids, including certain types of prostaglandins and thromboxanes, were elevated around parturition and decreased during the first week of lactation, whereas pro-resolving metabolites, such as resolvin D_2_ and 7-maresin 1, increased after parturition. Consistent with the results of the above-mentioned eicosanoids, the composite pro-inflammatory class was elevated at parturition and decreased thereafter, whereas the anti-inflammatory class changed in the opposite direction. Although the inflammatory state of postpartum dairy cows has been well documented [28], these are the first data to document an increase in anti-inflammatory eicosanoids during the resolution of inflammation as lactation proceeds. The causative role of eicosanoids in this resolution phase should be a fruitful area for further investigation.

Nuclear factor-κB (NF-κB) is a key transcription factor that plays crucial roles in inflammation [29]. In nonstimulated cells, NF-κB is bound to inhibitory IκB proteins and sequestered in the cytoplasm. Activation of NF-κB primarily occurs via the phosphorylation, ubiquitination, and degradation of IκB proteins; subsequently, the liberated NF-κB is translocated to the nucleus to drive expression of inflammatory genes [30]. One of the major IκB proteins in inhibiting NF-κB activation is IκBα, the abundance of which can be used to estimate anti-inflammatory status. In this study, hepatic IκBα abundance was significantly increased by rbTNFα in primiparous cows. This observation is unexpected, because TNFα is a potent activator of IκBα degradation [29], and suggests the involvement of compensatory anti-inflammatory adaptations in response to rbTNFα.

We also investigated the inflammatory mediator c-Jun, which is a key component of the pro-inflammatory transcription factor activator protein-1. The transcriptional activity of c-Jun is increased by phosphorylation of serine residues in response to various stimuli, including inflammation [31]. As for the NF-κB pathway, we found no evidence of hepatic c-Jun activation in response to rbTNFα. Collectively, rbTNFα treatment did not significantly alter the hepatic protein abundance of inflammatory mediators, but nevertheless greatly increased the plasma concentration of haptoglobin, an acute phase protein induced primarily by inflammatory signals in the liver [32]. It is possible that haptoglobin was primarily induced by transcription factors other than NF-κB or activator protein-1.

In this study, feed intake and milk production were decreased by rbTNFα treatments by 18 and 15%, respectively. TNFα is a key mediator causing anorexigenic responses in various diseases, and many of the effects of TNFα are dependent on its actions in the hypothalamus [33]. Interestingly, our previous research in late-lactation cows showed that 2 µg/kg rbTNFα treatment daily for 7 d also decreased feed intake by 18%, but did not significantly alter milk production [11]. Kushibiki et al. [25] reported that daily administration of 2.5 µg/kg rbTNFα for 7 d dramatically decreased feed intake (by ∼34%) and milk yield (by ∼15%). As expected, cows were in negative energy balance during the immediate postpartum period, indicating that their energy expenditure, driven largely by lactation requirements, greatly exceeded their energy intake. However, energy balance values were not affected by rbTNFα administration because the magnitude of decreased milk production was comparable to that of decreased feed intake.

Administration of rbTNFα did not alter plasma markers of lipid metabolism or promote fatty liver. As a lipolytic mediator in adipose tissue [34], previous studies found that rbTNFα administration at 2.5 µg/kg significantly increased plasma NEFA concentrations in mid-lactation cows [25] and heifers [35]. We also previously reported that 7-d rbTNFα administration at 2 µg/kg daily increased liver TG by ∼2-fold [11]. It is possible that because plasma NEFA and BHBA concentrations, as well as liver TG content, were already dramatically elevated in response to negative energy balance in the periparturient period, the doses of rbTNFα used in this study were insufficient to promote further alterations in lipid metabolism. On the transcriptional level, our previous work [11] in late-lactation cows showed that 2 µg/kg rbTNFα administration daily for 7 d increased the transcript abundance of *AGPAT1* (which catalyzes the esterification of a fatty acyl-CoA to the sn-2 position of the glycerol backbone in TG synthesis), tended to decrease *CPT1a* (which catalyzes transport of fatty acids into mitochondria for oxidation), and did not affect *ApoB* (which is a key component of very low-density lipoprotein that mediates hepatic TG exportation). Here, we did not find treatment effects for *AGPAT1* or *ApoB*, and found that rbTNFα increased *CPT1a* abundance dose-dependently. Collectively, rbTNFα did not promote the pathways favoring hepatic lipid accumulation in early-lactation cows.

Lactating cows rely heavily on hepatic gluconeogenesis to meet glucose requirements because the majority of dietary carbohydrates are fermented in the rumen. Under steady-state conditions, the rate of glucose appearance equals the rate of its disappearance, and the calculated glucose turnover rate approximately equals gluconeogenesis in ruminants, because net portal appearance of glucose is negligible [5]. In this study, there were no treatment effects on plasma glucose or insulin, abundance of key gluconeogenic transcipts, or plasma glucose turnover rate, indicating that low-grade inflammation did not affect hepatic gluconeogensis in early-lactation cows. Previous data regarding TNFα effects on gluconeogensis have been inconsistent. Short-term recombinant human TNFα administration at 10 µg/kg, but not 3.5 µg/kg, significantly increased glucose production (likely from glycogenolysis) and metabolic clearance rate in dogs; coupled with decreased fatty acid flux, the authors concluded that TNFα given in a high dose causes a shift toward carbohydrate as an energy substrate [36]. In a subsequent study, Sakurai et al. [37] infused 2.5 µg/kg TNFα in dogs for 2 h and did not observe a change in glucose production.

The primary substrate for gluconeogenesis in ruminants is propionate, a short-chain fatty acid derived from ruminal fermentation; as a result, liver glucose production in cows is often a function of energy intake [38]. To assess whether gluconeogenesis was affected by substrate supply, we expressed glucose production relative to feed intake. Interestingly, this analysis revealed that the 3 µg/kg rbTNFα treatment increased the apparent efficiency of gluconeogenesis from dietary substrates, primarily because of the low feed intake for that treatment. Given that propionate is used for glucose production at>90% efficiency in ruminants [39], increased metabolic efficiency was not likely the reason for this response. When requirements are greater than nutrient intake, such as in early lactation, the contributions of lactate, glycerol, and amino acids from body tissues to gluconeogenesis increase markedly [40]. Glycerol is released primarily from adipose TG mobilization, which was likely unaffected by treatments (based on unaltered plasma NEFA). In an attempt to evaluate whether 3 µg/kg rbTNFα-treated cows had greater amino acid supply from protein mobilization, we measured concentrations of plasma 3-methylhistidine, a marker of muscle protein breakdown that has been shown to increase rapidly during the periparturient period and peak at 1 week after parturition [41]. Treatment effects on plasma 3-methylhistidine were not significant; however, the 3 µg/kg rbTNFα treatment mean was numerically 20% greater than the other treatments. It seems likely that a combination of slightly enhanced muscle proteolysis and decreased amino acid use for milk protein synthesis resulted in greater supply of amino acids for gluconeogenic substrate in the 3 µg/kg rbTNFα treatment.

Despite the lack of dramatic alterations in metabolism, the higher dose of rbTNFα tended to impair the health of postpartum cows. Health disorders affect considerable numbers of periparturient dairy cows, compromising animal welfare, milk production, and fertility. Ketosis is considered the most important metabolic disease in the U.S. dairy industry [3], and ketotic cows are also at increased risk for other disorders, such as displaced abomasum and metritis [42]. Ketosis may increase the risk of infectious disorders through negative effects of ketone bodies on immune cell function [43]. In this study, rbTNFα increased the incidence of ketosis by 3-fold in the first week of lactation, accounting for much of the increase in total disease incidence. There are numerous mechanisms by which rbTNFα may have promoted subclinical ketosis, but poor feed intake in early lactation is a key risk factor for ketosis [1], and it is likely that the suppression of intake by rbTNFα played a role in this study. Decreased feed intake did not decrease energy balance or increase lipolysis in this study, but low intake affects more than just energy status; decreased protein, mineral, vitamin, or lipid intake may have adversely affected health.

## Conclusions

In conclusion, rbTNFα treatment during the first 7 d of lactation decreased feed intake, reduced milk production, and tended to impair health status, but did not significantly alter glucose or lipid metabolism. These data indicate that in response to low-grade inflammation, early-lactation cows compensate for the reduced intake by suppressing milk production rather than further compromising their energy balance or systemic metabolism. This coordinated physiological response to inflammatory mediators can be considered a biological tradeoff between lactational performance and survival [44]. However, because effects on feed intake and milk yield occurred simultaneously, it is unclear whether one or the other was a secondary response. Further studies are needed to investigate if inflammation directly alters mammary or neurological function. Nevertheless, this study indicates that preventing excessive inflammation in early lactation has the potential to improve productivity and health of dairy cows.
